# Guidelines for measuring and reporting environmental parameters for experiments in greenhouses

**DOI:** 10.1186/s13007-015-0083-5

**Published:** 2015-09-11

**Authors:** A J Both, L Benjamin, J Franklin, G Holroyd, L D Incoll, M G Lefsrud, G Pitkin

**Affiliations:** NCERA-101, Department of Environmental Sciences, Rutgers University, New Brunswick, NJ USA; Rothamsted Research, Harpenden, Hertfordshire AL5 2JQ UK; CEUG, University of Lancaster, Lancaster, LA1 4YQ UK; Department of Plant Sciences, University of Leeds, Leeds, UK; NCERA-101, Department of Bioresource Engineering, McGill University, Montreal, Canada; CEUG, The James Hutton Institute, Dundee, AB15 8QH Scotland

**Keywords:** Science communication, Data sharing, Quality assurance, International standards

## Abstract

**Background:**

The importance of appropriate, accurate measurement and reporting of environmental parameters in plant sciences is a significant aspect of quality assurance for all researchers and their research. There is a clear need for ensuring research across the world can be compared, understood and where necessary replicated by fellow researchers. A common set of guidelines to educate, assist and encourage comparativeness is of great importance. On the other hand, the level of effort and attention to detail by an individual researcher should be commensurate with the particular research being conducted. For example, a researcher focusing on interactions of light and temperature should measure all relevant parameters and report a measurement summary that includes sufficient detail allowing for replication. Such detail may be less relevant when the impact of environmental parameters on plant growth and development is not the main research focus. However, it should be noted that the environmental experience of a plant during production can have significant impact when subsequent experiments investigate plants at a molecular, biochemical or genetic level or where species interactions are considered. Thus, researchers are encouraged to make a critical assessment of what parameters are of primary importance in their research and these parameters should be measured and reported.

**Content:**

This paper brings together a collection of parameters that the authors, as members of International Committee on Controlled Environment Guidelines (ICCEG) in consultation with members of our three parent organizations, believe constitute those which should be recorded and reported when publishing scientific data from experiments in greenhouses. It provides recommendations to end users on when, how and where these parameters should be measured along with the appropriate internationally standardized units that should be used.

## Background

Research greenhouses are important tools for scientists and engineers studying the effects of environmental parameters on the growth and development of a large range of agronomic and ornamental plant species. In addition, the greenhouse environment allows for research to continue year-round so that external environmental influences have minimal effect on the experimental set points. The design and operation of research greenhouse facilities vary significantly from one facility to the next [1–4]. Therefore, accurate environmental control and data collection are essential to ensure that experiments can be adequately evaluated by research colleagues and replicated among different facilities [5].

The paper builds on two previous ICCEG guidelines covering minimum sets of parameters that should be reported when working in plant growth rooms and chambers [6] and in tissue culture facilities [7]. The focus on greenhouses in this publication is complemented by earlier works relating to plant growth chambers [8, 9].

It is the researcher’s responsibility to become familiar with the operational principles of the sensor(s) used during experimentation. The principle of operation, the manufacturer’s design of the sensor, and the specific sensor installation can all have an effect on the readings collected. These effects should be understood and considered when evaluating the measurements. Due to the spatial and temporal variability of many environmental parameters in greenhouse environments, it is important to carefully consider sensor placement, method of data processing, and the impact this variability may have on the experimental conclusions presented.

When reporting experimental results, it is recommended that the equipment and instrument information (model, manufacturer, town, country) is included, including information on precision and accuracy, when it is of specific importance to the experiment(s) conducted. In addition, the calibration procedure and the time of the most recent calibration should be reported. It is good practice to calibrate sensors before and after experiments so that any changes in calibration can be evaluated and incorporated into the data analysis. Periodic factory re-calibration of sensors is recommended (follow manufacturers’ recommendations), and proper calibration can be verified by comparing sensor output with the output from calibrated sensors that are otherwise stored and only used for the purpose of calibration. Note that such calibration sensors may require periodic re-calibration also. In all cases, multi-point calibration of sensors is recommended.

These guidelines describe and discuss environmental parameters that affect plant growth and development. These parameters are divided into five categories: radiation, temperature, gases, water, and nutrients that are each discussed. In addition, greenhouse structure, growing system and environmental control system/strategies are included as a sixth comprehensive parameter. Greenhouse designers, managers and researchers conducting plant experiments in greenhouses are encouraged to review and implement the monitoring and reporting practices discussed in these guidelines. The guidelines conclude with a sample report describing how the environmental conditions during an experiment should be reported in the literature.

## Compliance and quality assurance

Whether growing conventional plants, genetically modified organisms (GMOs) or quarantine crops, producers (including those operating in research facilities) need to comply with an increasing range of regulations, codes of practice, legislation and/or standards. For example, compliance is often needed regarding hazardous material and waste management and/or environmental and health and safety regulations such as the Australian/New Zealand Standard [10]. Other regions of the world may have similar compliance requirements that need to be followed. In addition, sponsors of research increasingly expect a minimum standard of scientific practice, as described for example in the Research Councils’ United Kingdom Policy and Guidelines on Governance of Good Research Conduct [11]. It specifies ‘weak procedures, inadequate documentation of procedures or inadequate record-keeping’ are inappropriate research behaviour. Furthermore in the UK, many grants by Research Councils and other government departments are made conditional on abiding by the Joint Code of Practice for Research [12] that includes the statement ‘All equipment must be appropriate for the measurements to be made, calibrated at the appropriate interval, and be in good working condition’.

For greenhouse experiments, there are no standards that are equivalent to the International Organization for Standardization (ISO) ISO/IEC 17025 used for testing and calibration laboratories [13]. However, some of the practices that laboratories adopt to meet this standard’s requirements could be beneficial for research in greenhouse facilities. Although they may appear daunting at first, they can be implemented with little effort and give rewards in terms of more effective operation. These include documenting methods (i.e. calibration and measurement), listing all staff competent in those methods, records of their training, and identification of sensors and equipment. For each item of measurement equipment there should be a log book recording when and how it was calibrated, who undertook the calibration and records of any observations on performance. Annotated records of all measurements should be stored in multiple locations.

It is recommended to utilise an independent set of sensors to measure environmental parameters in greenhouse environments, separate from those sensors used for monitoring and control. The reasons are:

- Sensors used for monitoring and control may not have the same specifications and/or operational requirements as the ones recommended for experimental measurements (e.g. response time, resolution, physical location).
- Even if sensors used for monitoring and control are serviced and calibrated, they may still malfunction during an experiment. Therefore, an independent set of sensors provides added assurance.
- Many sensors include, in addition to the actual probe, electronics and software that interpret and in some cases display the signals. Using different probes in the same hardware package does not offer truly independent assurance.
- Some regulatory bodies require that experiments are conducted using monitoring where the data recording the environmental conditions have assured integrity. This integrity includes not allowing any of the raw data collected to be altered by the user. Systems to monitor and control the greenhouse environment may not allow such a level of data integrity.

## Definitions

### Radiation

- Irradiance: The incident radiant energy flux per unit plane surface area (J m^−2^ s^−1^ or W m^−2^).
- Photon: The smallest packet (‘particle’) of electromagnetic radiation having no mass, and no electrical charge.
- Photon irradiance: The incident photon flux per unit plane surface area (also known as photon flux density; µmol m^−2^ s^−1^).
- Photosynthetically active radiation (PAR): The part of the radiation spectrum that is by definition used by plants for photosynthesis (between 400 and 700 nm; µmol m^−2^ s^−1^).
- Radiation integral: The radiation flux summed over a period of time (typical units for plant experiments: mol m^−2^ d^−1^).
- Red:far red (R:FR) ratio: The red to far red ratio (ratio of radiation emitted at or close to 660 and 730 nm).
- Solar radiation (short wave radiation): The radiation emitted by the Sun (approximately between 280 and 3,000 nm; W m^−2^).
- Spectrum: The distribution of energy (light) emitted by a radiation source in relation to wavelength.
- Waveband: A consecutive portion of the radiation spectrum.
  - 100–380 nm Ultra violet (UV)
    1. 100–280 nm UVC
    2. 280–315 nm UVB
    3. 315–380 nm UVA
  - 380–770 nm visible
  - 400–700 nm PAR
  - 770–3000 nm near infrared (NIR)
  - 3000 nm–1 mm far infrared (Long wave)
- Wavelength: The distance between one peak (crest) of a wave of light and the next corresponding peak (nm).

### Temperature

- Aspirated sensor: A sensor placed in an enclosure equipped with a downstream fan to provide continuous air movement around the sensor (with an air speed of approximately 3 m s^−1^). The sensor should be placed at a representative location near the crop canopy.
- Exposed sensor: A sensor fully exposed to the environment.
- Shielded sensor: A sensor shielded from solar radiation but exposed to passive air movement.
- Surface temperature: The temperature of the outer surface of an object. An object’s surface temperature is one of the parameters that determine how much energy it radiates to its surroundings (Stefan–Boltzmann Law) (°C).

### Gaseous concentration in air

#### Water vapour in air

- Dew point temperature: The temperature to which the air must be cooled before the water vapour contained in the air begins to condense (*T*_*dp*_) (°C).
- Dry bulb temperature: The temperature measured by a thermometer freely exposed to air but shielded from radiation and moisture (*T*_*db*_) (°C).
- Humidity ratio: The ratio of the mass of water vapour to a unit mass of dry air (containing both water vapour and other gaseous constituents) (*w*) (kg/kg)
- Partial vapour pressure: The pressure water vapour would exert if it alone occupied a volume of air (i.e. excluding the pressure resulting from other constituents of air) (*e*) (Pa).
- Relative humidity: The ratio of the partial vapour pressure (*e*) to the saturated vapour pressure (*e*_*sat*_) at the dry bulb temperature (*RH*) (%).
- Vapour pressure deficit: The difference between the pressure exerted by water vapour in a volume of air and the pressure that would be exerted by the water vapour that volume could hold if the air was saturated (*VPD*) (kPa).
- Wet bulb temperature: The lowest temperature that can be measured after adiabatic evaporation of pure water from a wick placed around an aspirated thermometer bulb (*T*_*wb*_) (°C).

#### Carbon dioxide in air

- Atmospheric CO_2_ concentration: The concentration of carbon dioxide molecules in a mixture of air [*CO*_*2*_] (µmol mol^−1^).

#### Oxygen in aqueous solution

- Dissolved oxygen concentration: The concentration of oxygen molecules in an aqueous solution [*O*_*2*_] (mg L^−1^).

### Liquid water

- Volumetric water (or moisture) content: The volume of water contained in a unit volume of soil. Widely used and measured with a variety of soil moisture sensors (*θ*_*v*_) (dimensionless fraction).
- Gravimetric water (or moisture) content: The mass of water in a unit mass of soil. Determined by weighing the soil before and after drying at 105°C (*θ*_*m.*_) (dimensionless fraction).
- Matric potential: The tension (suction) in the growing media that arises from the interaction between water and the matrix of solid particles (*ψ*_*m*_) (kPa).

### Nutrients

- Nutrient concentration: The concentration of an inorganic element in liquid solution or in a growing medium [*element*] (mmol L^−1^, mol kg^−1^ or g L^−1^).

## Recommended instruments and sensors

### Radiation

The choice of radiation sensor depends on whether it is the energy content or photon content that is the quantity of interest [14] (Table 1).

**Table 1:** Instruments and sensors for measurement and their calibration

What to measure	Units	Measured by	Precision of instrument^b^	Accuracy of reading^b^	Calibrated by and when
Radiation (PAR^a^)	µmol m^−2^ s^−1^	Quantum sensor	±1 %	±10 %	Comparison with a reference sensor or against a standard quartz-halogen lamp traceable to a (inter)national standard (e.g. US NIST). Once per annum
Radiation (net)	W m^−2^	Net radiometer	±2 %	±5 %	Comparison with a reference meter or in a temperature controlled calibration chamber capable of evaluating short and long wavebands. Once per annum
Radiation (spectral)	µmol m^−2^ s^−1^ nm^−1^	Spectroradiometer	±1 %	±5 %	Comparison with a reference meter or against a standard source of radiation (e.g. ASTM G138-06). Once per annum
Irradiance (solar)	W m^−2^	Pyranometer	±1 %	±5 %	Comparison with a reference meter or against a standard source (traceable to the World Radiometric Reference) in an integrating sphere. Once per annum
Radiation (integral)	MJ m^−2^ d^−1^ or mol m^−2^ d^−1^	Calculated from accumulated radiation data	–	–	–
Air temperature	°C	RTD, thermocouple or thermistor (shaded and aspirated in air with speed ≥3 m s^−1^)	±0.1 °C	±0.2 °C	Comparison with a reference thermometer (e.g. traceable against US NIST) by placing in melting crushed ice and boiling distilled water. Once per annum
Substrate temperature	°C	RTD, thermocouple or thermistor (ensure good contact with substrate)	±0.1 °C	±0.2 °C	As above
Surface temperature	°C	Infrared temperature sensor, fine wire thermocouple	±0.1 °C	±0.2 °C	Infrared sensor: Comparison with a reference surface thermometer mounted on the same surface within the field of view of the infrared sensor Thermocouple: as above
Atmospheric moisture: relative humidity or vapour pressure deficit (VPD)	% or kPa	Capacitance, dewpoint sensor, psychrometer, or IRGA (infrared gas analyser)	Relative humidity: ±2 % Dewpoint temp.: ±0.1 °C VPD: ±0.3 kPa	±5 % ±0.5 °C ±0.5 kPa	Humidity generator; unsaturated salt solution calibration standards (35 and 80% RH). Once per annum
Air speed	m s^−1^	Anemometer (range 0.1–15.0 m s^−1^)	±2 %	±5 %	Wind tunnel. Once per annum
pH	–	pH probe (range 3–10)	±0.1 pH	±0.1 pH	Standard solutions. Before every measurement, or weekly in continuous measurement applications
Electrical conductivity (EC)	S m^−1^	Electrical conductivity meter	±3 %	±5 %	As above
Dissolved oxygen	mg L^−1^	Dissolved oxygen meter (maintain adequate solution flow rate and ensure temperature compensation)	±3 %	±5 %	As above
Atmospheric CO_2_ concentration	µmol mol^−1^	Silicon based NDIR (non-dispersive infrared) sensor as part of an IRGA (infrared gas analyser)	±1 %	±3 %	Certified calibration gases for low and high end of the measurement range, and/or precision gas mixing instrument. Once per week

^**a**^Referred to as photosynthetically active radiation (PAR: 400–700 nm) for general usage and described as photosynthetic photon flux density (PPFD) by many journals, professional societies and manufacturers of quantum sensors.

^**b**^Precision is how close the measured values are to each other. Accuracy is how close a measured value is to the actual (true) value.

**Fig. 1:**
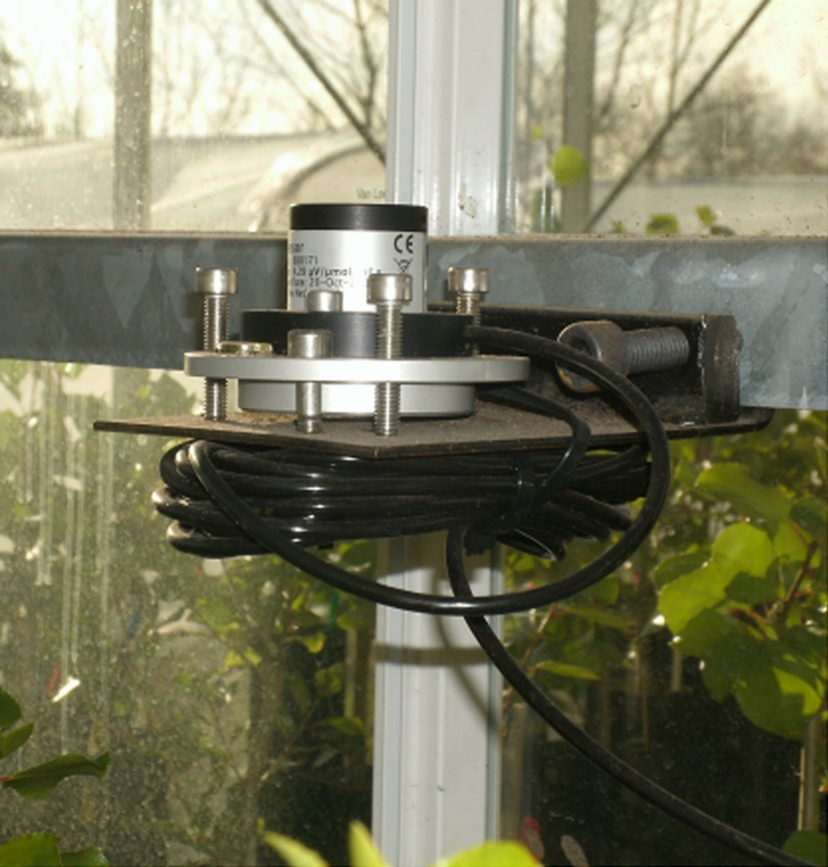
A quantum sensor on a leveling platform mounted at the top of the canopy for measuring photosynthetically active radiation (PAR). Radiation sensors should be chosen for the specific purpose required. Positioning should be carefully considered to avoid shading or noise from reflective surfaces.
- Spectroradiometer: Measures the flux in discrete wavelength intervals across a specific waveband. This sensor is recommended when evaluating the radiation emitted from LED lighting systems because the narrow waveband emitted by LED sources is not always accurately measured by other radiation sensors.

- Net radiometer: Measures the difference between the incoming and outgoing radiation in W m^−2^. This sensor is typically used to evaluate the radiant energy environment in the greenhouse.
- Pyranometer: Measures total solar radiation in W m^−2^. This sensor is typically used to evaluate the incoming radiant energy from the sun.
- Quantum sensor: Measures photosynthetically active radiation in µmol (photons) m^−2^ s^−1^ (Fig. 1).

### Temperature

- Infrared thermometer: A non-contact sensor appropriate for measuring surface (e.g. leaf) temperature. Infrared thermometers typically have a conical field of view that depends on sensor make and model, and distance from the surface being measured.
- Resistance temperature device (RTD): A sensor that operates on the principle that the electrical resistance in a thin wire or film is a function of temperature. The thin wire or film are often made of platinum and encapsulated in another material (e.g. ceramics or glass).
- Thermistor: A sensor that operates on the principle that the change in electrical resistance in a ceramic or polymeric material is a function of temperature. Thermistors typically achieve a higher precision across a more limited temperature range than RTDs.
- Thermocouple: A sensor made of two wires of dissimilar metals, fused at one end to form a junction, that produce a voltage, which is a function of the temperature difference between the junction and the other end of the two wires. An accurate reference temperature measurement is needed at the point where these two wires are connected to the meter (terminal).

### Gases

#### Water vapour

**Fig. 2:**
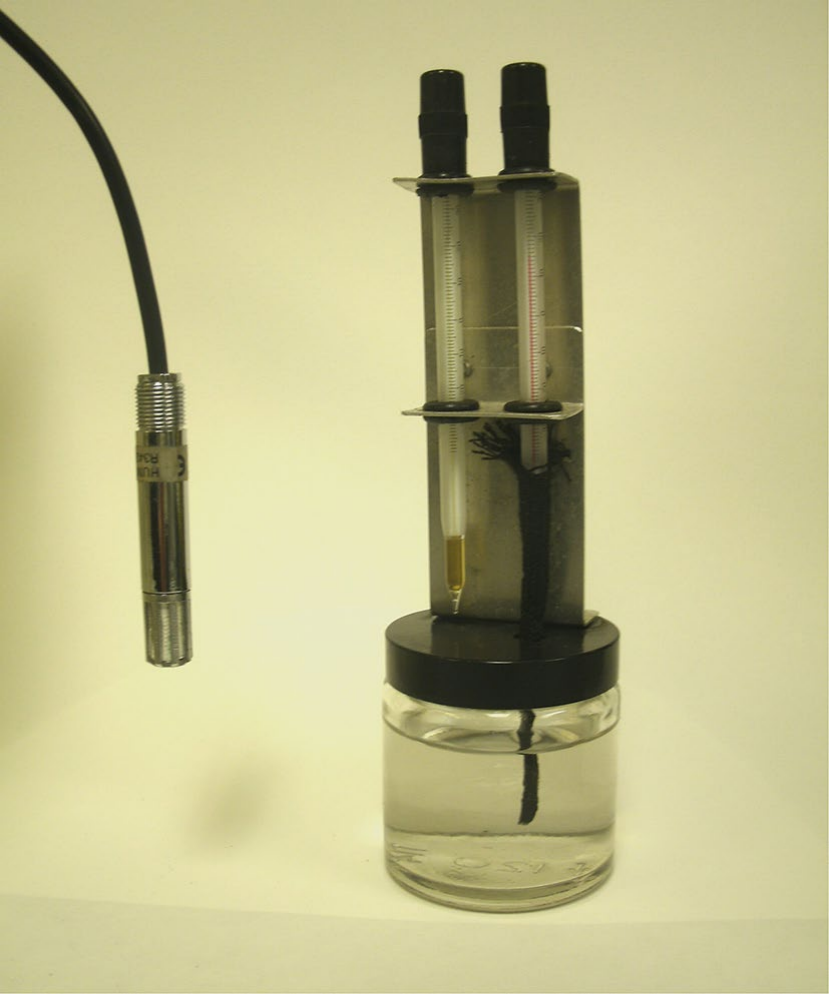
Water vapour (humidity) sensors may be relatively inexpensive capacitance hygrometers (*left*) or a basic psychrometer (*right*).
- Dew point hygrometer (condensation hygrometer): A sensor that measures the scattering of light reflected from a mirror that is chilled to the point when condensation occurs. These dewpoint temperature sensors are expensive and require frequent cleaning because contamination on the mirror can cause errors. The measurements are precise (±0.1°C) over a wide range (−100 to +100°C). The measurement process does not distinguish between the formation of ice or dew on the mirror.
- Psychrometer: A device that consists of two matched thermometers over which air is drawn (Fig. 2). One of the thermometers has its bulb covered with a wetted (with pure water) cloth or wick. These so-called wet and dry bulb sensors are relatively cheap, have good stability and tolerate condensation without damage. Accuracy ranges from ±2 to ±5% of true value and is affected by contamination (dirt or deposits) on the cloth/wick. The atmospheric humidity typically must be calculated using psychrometric equations, or read from a psychrometric table (Mollier diagram).

- Capacitance hygrometer: A sensor that measures the resistive or capacitive changes in materials (Fig. 2). A capacitive humidity sensor consists of a thin-film polymer sandwiched between two electrodes that record its capacitance. These sensors are inexpensive, small and require little maintenance. They have a wide measurement range (0–100%), a wide temperature range, high stability, fast response time, fully recover from condensation and are highly resistant to contamination. These sensors lose accuracy at low humidity (<5%) and require calculations (often accomplished using electronics) to convert capacitance to relative humidity.

#### Carbon dioxide

- Non-dispersive infrared (NDIR) CO_2_ sensor: Carbon dioxide absorbs infrared radiation between 4,000 and 4,500 nm. This absorption can be used to measure the volumetric CO_2_ concentration with non-dispersive infrared (NDIR) CO_2_ sensors, typically in an IRGA (infrared gas analyser). Since water vapour also absorbs infrared radiation, particularly in the 2,500–3,000 nm waveband, it is important to either remove the water vapour before measurement (by drying for example with a desiccant), adjust the sensor reading to account for the presence of water vapour, or alternately, install a pass-filter (interferometer) in front of the IR detector to ensure that only wavelengths that are absorbed by CO_2_ are able to reach the detector. Zero calibration is performed by supplying the sensor with pure nitrogen gas. Additional calibration can then be performed using a calibration gas with a known CO_2_ concentration at the high end of the measurement range. When air samples are drawn from (warm) greenhouse sections and pumped to a (colder) CO_2_ sensor located outside the greenhouse, care should be taken to avoid condensation in the sampling tube, pumping system, and sensor components.

#### Dissolved oxygen

- Dissolved oxygen meter: The dissolved oxygen (DO) concentration in a nutrient solution is most easily measured with a DO meter. The sensing component of a DO meter, a polarographic probe, consists of an anode and a cathode submerged in an electrolyte (e.g. potassium chloride) that is held in place by a semi-permeable membrane. Very small DO sensors consume very little oxygen, removing the need to stir the solution. A DO probe requires special care: the membrane must be kept wet and the probe filled with electrolyte. The electrodes can be cleaned by dipping them in ammonium hydroxide. The meter must be calibrated regularly (e.g. using a two-point calibration of 0 and 100% air saturated water).

### Liquid water

For properties of rooting medium and/or irrigation water:

- Dissolved oxygen meter (see “Dissolved oxygen”).
- Electrical conductivity (EC) meter: A sensor that measures the electrical conductivity of a nutrient solution based on the total amount of dissolved salts (nutrients) and the resulting ion concentration. In solutions with ions from multiple elements, this sensor is not able to measure the concentration of individual elements. The EC measurement provides an indication of overall nutrient concentration in a solution. If a measurement of individual elements is required, ion-selective sensors can be used (see “Nutrients”). EC meters are inexpensive and can be calibrated with commercially available calibration solutions. EC measurements are often combined with pH measurements for a quick and easy assessment of root zone or irrigation water.
- Soil/media/liquid temperature sensor (see “Temperature”).
- Stem psychrometer: A sensors that measures the stem water potential in situ of woody plant species. A small measurement chamber is clamped onto a section of exposed sapwood. Two thermocouples measure the wet bulb depression between the temperature of the sapwood and the temperature inside the measurement chamber. Using temperature corrections, the stem water potential can be accurately determined. This measurement integrates the impact of all environmental conditions on plant status. Time series measurements can reveal the degree and changes of plant water stress.
- Tensiometer: A sensor that measures soil water tension. Soil water tension is equal to the tension (force) the root system must overcome to extract water from the soil. The sensor measures the tension (suction) on an enclosed water reservoir that is in continuous contact with the soil matrix through a porous ceramic cup. The less water in the surrounding soil, the higher the suction on the water reservoir.
- Soil moisture sensor: A sensor that measures the volumetric water content of the root zone. These sensors determine the electrical resistance or the dielectric constant as a proxy for moisture content. Sensor types include capacitance (frequency domain) or time domain reflectometry sensors. As tensiometers, these sensors can be used to manage crop irrigation cycles.

### Nutrients

- Weighing scale: In addition to the sensors described above (see “Liquid water”), a scale with sufficient accuracy (e.g. 0.01 g or ±1 display resolution) should be used to weigh the proper amount of fertiliser (containing one or more nutrients). The frequency of calibration of the scale should be recorded.
- While some ion-selective sensors are beginning to become available, they are not yet widely used because of concerns over price, stability and longevity. This may change in the future. However, if these sensors are used, make and model should be reported as well as accuracy and frequency of calibration.

## Parameters to be monitored and recorded

**Fig. 3:**
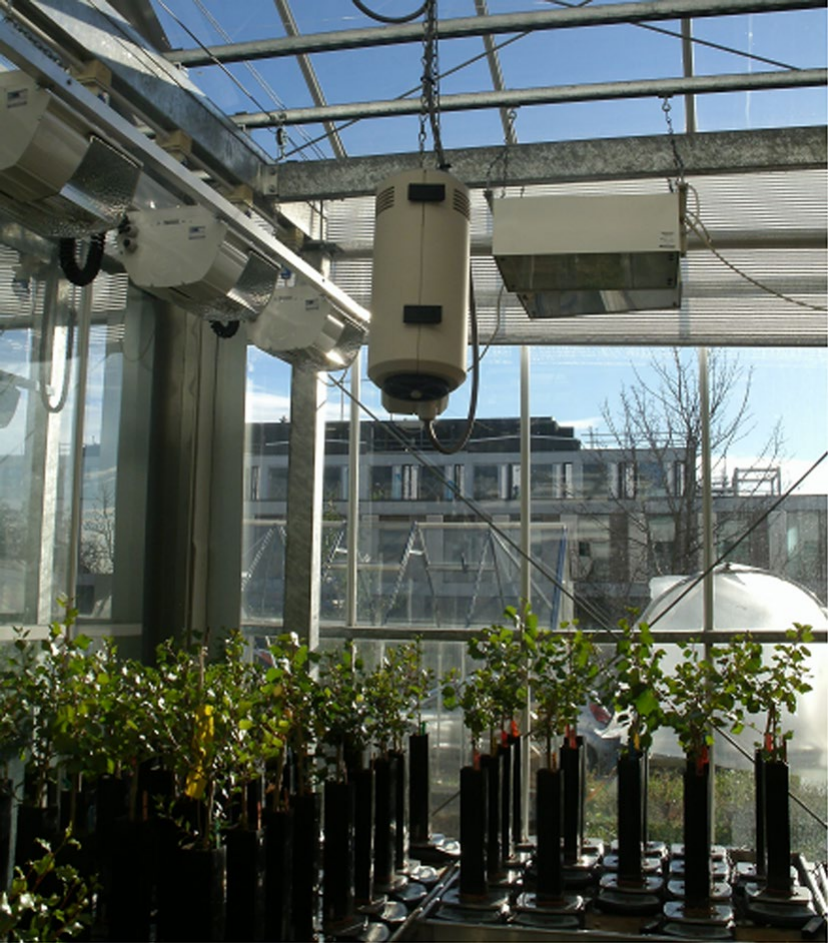
An aspirated sensor box suspended centrally in free air for recording environmental parameters. The sensors are located inside the *box* while air is constantly drawn through the *box*. Sensor (*box*) type and location along with frequency of sampling and details of data integration should be reported.

Due to spatial and temporal fluctuations in the greenhouse environment, all measurements should be taken in as many locations as practically feasible and as frequently as possible, preferably continuously. When using a data logging system, the measurement frequency can be higher than the recording frequency. In that case, it is important to report what type of processing was used (e.g. scanning and averaging intervals) to record the data. The following points should be reported: (1) sensor location(s) or sampling point(s), whether aspirated or shielded (Fig. 3), (2) average and standard deviation of the measurements, and (3) method and frequency of sensor calibration (Table 2).

**Table 2:** Primary parameters

What to measure	Units	Where to measure	When to measure	What to report
(a) Minimum set				
Air temperature^a^	^o^C	At canopy level, in centre of growing area. Location of sensor is crucial and should be independent of the greenhouse temperature control sensor. Include another sensor for outside temperature	Preferably continuous, but at least hourly	Mean and standard deviation for light and dark periods. Number of locations (preferably more than one)
Substrate temperature	^o^C	Centre of root matrix for solid and liquid substrates	As above	As above
Radiation (PAR^a^)	µmol m^−2^ s^−1^	At top of canopy, in centre of growing area	Preferably continuous but at least hourly	Mean and standard deviation. Number of measurement locations (preferably more than one). When used, supplementary radiation sources (type, model and manufacturer, distribution, energy consumption, conversion efficiency), and their duration of operation
Photoperiod	h	–	Daily or when conditions change	Duration of light period (including any night interruption)
Atmospheric moisture: relative humidity or vapour pressure deficit (VPD)	% or kPa	At canopy level, in centre of growing area and independently of the greenhouse humidity control sensor	Preferably continuous but at least hourly	Mean and standard deviation for light and dark periods. Number of locations (preferably more than one)
Atmospheric CO_2_ concentration^b^	µmol mol^−1^	At canopy level, at a representative location	Preferably continuous but at least hourly if CO_2_ enrichment is used	Mean and standard deviation/Number of measurement points and their location relative to the plant canopy
pH	–	In root zone environment, or in nutrient solution directly applied to the root zone environment	Preferably continuous, but at least hourly	Mean and standard deviation, Location of measurement(s)
Electrical conductivity (EC)	S m^−1^	In root zone environment, or in nutrient solution directly applied to the root zone environment	Preferably continuous, but at least hourly	Mean and standard deviation, Location of measurement(s)
Nutrient—liquid media	mmol L^−1^	–	Daily or when replenished	Ionic concentration in added solution. Frequency of additions. Aeration if any
Nutrient—solid media	mol kg^−1^ (dry)	–	When added or replenished	Nutrients and their form added to soil media. Frequency of additions
Watering	Litre (L)	Growing system	At start of experiment and when changed	Frequency, amount, duration and type of water added per unit area or per plant. Mean and standard deviation. Type of irrigation system
Plant alignment	–	On bench/floor/hanging system	At start of experiment and when conditions change	Number of plants per unit area and number of re-spacings or relocations
Greenhouse properties		Greenhouse	At start of experiment	Latitude and longitude. Orientation of long axis relative to compass North. Size (floor area m^2^, growing area m^2^, gutter height m, peak height m), type (free standing, gutter connected), shape (curved roof, peaked roof). Manufacturer and model if available, indicate if it has special features (e.g. type of glazing material, energy/shade curtain)
(b) Additional parameters				
Surface temperature ^b^	^o^C	Plant tissue: pointed at canopy or individual leaf surface. Greenhouse: pointed at structural surface	Preferably continuous, but at least hourly	Mean and standard deviation. Location and orientation of the sensor. Distance of the sensor to the surface measured. Field of view of the sensor
Radiation (net)	W m^−2^	At top of canopy, in centre of growing area	As above	Mean and standard deviation. If measured, also report solar radiation so that (net) long wave radiation can be determined
Radiation (spectral)	µmol m^−2^ s^−1^ nm^−1^	As above and/or where of interest (e.g. within the canopy)	As often as practical	Mean and standard deviation. Absolute or relative contribution of a specific wavelength or waveband to the overall radiation
Irradiance (solar) W m^−2^	W m^−2^	Outside (unobstructed) and/or inside (at top of canopy, in centre of growing area)	Preferably continuous, but at least hourly	Mean and standard deviation. When possible, calculate (average) transmission of radiation through the greenhouse cover
Radiation (integral)	MJ m^−2^ d^−1^ or mol m^−2^ d^−1^	Calculated from accumulated data	Continuously during the measurement interval	Accumulated (typically daily) values. Relative contributions of supplementary and solar radiation to (daily) integral
Air circulation	m s^−1^	At canopy level	At start of experiment and more frequently if conditions change	Mean and standard deviation Design of circulation system. Predominant direction of flow. Number of measurement points and their location relative to the plant canopy. Report whether open or closed greenhouse
Substrate water content: volumetric or gravimetric water content, or matric potential	% or kPa	Substrate	Daily	Mean and standard deviation. Number of measurement locations
Dissolved oxygen	mg L^−1^	In root zone environment, or in nutrient solution directly applied to the root zone environment	Preferably continuous, but at least hourly	Mean and standard deviation. Location of measurement(s)

^**a**^Referred to as photosynthetically active radiation (PAR: 400–700 nm) for general usage and described as photosynthetic photon flux density (PPFD) by many journals, professional societies and manufacturers of quantum sensors.

^**b**^Report if records are available, and always when it is a variable under investigation.

Table 2 lists two sets of parameters. The minimum set comprises the recommended basic set of parameters that should be measured and recorded during the course of most experiments and reported in publications. The additional set includes parameters that could be measured and reported provided appropriate sensors are available and the parameters are pertinent to the type of experiment conducted. It also includes parameters that will comprise metadata (describing content and context of stored data files) held by facility managers and available to users if required.

### Radiation

#### Measurements

Since the human eye is an inaccurate sensing device for quantifying light, sensors are needed to measure the radiation environment inside greenhouses that is constantly fluctuating due to daily and seasonal changes in solar altitude, degree of cloud cover, and shadows resulting from structural components and overhead equipment [15, 16]. In addition, the radiation flux and spectrum changes when measured at different locations within a plant canopy. Unless light transmission in a plant canopy is the focus of research, PAR and its integral should be measured at the top of the plant canopy while assuring that no parts of the plant canopy shade the sensor. The location of the top of the plant canopy should be referenced relative to a fixed object in the greenhouse (e.g. the floor or the bottom of the supplemental light fixtures).

When a combination of sunlight and supplementary light is used to grow a crop, it is important to quantify how much of the total light was provided by each source. If this is not done, it is virtually impossible to repeat the experiment under similar conditions. Solar irradiance can be modified as it passes through greenhouse cladding material and/or interacts with the plant canopy. As a result, changes in the amount of UV and/or the R:FR ratio can occur and these should be monitored and reported when they have an impact on plant growth and/or development.

When conducting supplementary or photoperiod lighting experiments, researchers need to be aware of the impact these treatments can have from unintended spillage of light into neighbouring greenhouse sections. Curtains may need to be used to shield experimental sections.

Additional outside radiation sensors are often used to compare the radiation environments inside and outside the greenhouse. This comparison provides information about the transmission of radiation through the greenhouse structure. Outside radiation can also be used as input for the greenhouse control strategy [15, 16].

#### Light sources

To supplement sunlight, typical greenhouse radiation sources include: high-pressure sodium (HPS), metal halide (MH), incandescent (INC), fluorescent (FL) lamps, and light emitting diodes (LED). Each radiation source has its own unique emission spectrum that can result in unique plant responses [17]. Therefore it is important to report the type of supplementary radiation source that was used, what its spectral output was (particularly when unusual) and the mix (%) by wattage of any mixed source. It is also important to report the duration of the supplemental lighting period.

#### Effect on plant growth

The rate of photosynthesis is by definition dependent on the amount of photosynthetically active radiation (PAR) absorbed by the plant canopy. While measuring PAR, researchers should be mindful of the light compensation point (where the net rate of photosynthesis becomes positive) and the saturation level associated with the environmental conditions and the plant species under investigation [15, 17]. In most cases, it is useful to report the relative contributions of sunlight and supplementary light to daily light integral achieved.

#### Effect on plant development

For many plant species, especially ornamentals, changes in daylength can trigger physiological responses (e.g. flower initiation). Therefore, it is important to report the length of the photoperiod imposed as well as the method used to implement the photoperiod (e.g. with black-out curtain or with supplementary lighting). When extending the natural daylength or providing night interruption, the light source, its radiation flux and spectrum, as well as the start time and duration of the lighting period should be reported, especially if it is different from the source of supplementary light for growth.

#### Effect on plant morphogenesis

For some plant species, spectral characteristics of radiation can affect morphology (e.g. internode elongation, flower rotation towards the sun). When these morphological changes have an effect on growth and development, the responsible spectral characteristics should be quantified and reported.

### Temperature

#### Measurements

The greenhouse air temperature is determined by outside temperature, incoming and outgoing radiation, radiative, conductive and convective heat transfer associated with the structure, and the operation of the greenhouse heating and cooling system. Significant spatial and temporal fluctuations can result from daily or seasonal changes in outside conditions. As for radiation measurements, structural elements can generate shadows that can (temporarily) affect temperature measurements. In addition, these fluctuations can be the result of the management strategy used to maintain indoor conditions, including the heating, ventilation and cooling system as well as internal air circulation systems such as horizontal airflow fans or perforated polyethylene tubing distribution systems (poly-tube). During ventilation and cooling (especially when using an evaporative cooling pad or fog cooling), it is common to experience significant temperature gradients from the air inlet to the outlet. Minimizing these gradients requires understanding of the airflow patterns resulting from the ventilation system and/or additional internal air recirculation. As a result, continuous measurements, preferable at multiple locations, are needed to assess the average temperature conditions experienced by the plants. If only one measurement location can be monitored, care should be taken in selecting the most appropriate location for sensor placement (typically near the canopy in the centre of the growing area).

Maintaining optimum root zone temperatures can be critical for timely and/or maximum production of many plant species. For those plant species, root zone temperatures should be measured and recorded in at least one, but preferably more, representative location(s). Vertical temperature gradients can be expected and are typically more significant when plants are grown on the floor, whether that floor is heated or not. For hydroponic systems, the temperature of the (recirculating) nutrient solution should be reported.

#### Effect on plant growth

The rates of chemical reactions typically increase with temperature. Within plants, most chemical reactions, including photosynthesis, are enzyme driven. Hence, the rate of reaction often decreases above an optimum because high temperatures damage the enzymes. Many plants produce compounds to protect against high and low temperatures. On the other hand, in some plants species extreme temperatures can result in abnormal morphological characteristics. In some cases, it is useful to record not just the average temperature and standard deviation, but also the maximum and minimum values the plants were exposed to. For the production of field crops (e.g. corn—*Zea mays* L.) in greenhouses, the calculation of the crop heat units (growing degree days and associated base temperature) should be reported.

#### Effect on plant development

For many plant species, especially ornamentals, changes in temperature can trigger physiological responses (e.g. flower initiation). For those plant species, it is important to report the maximum temperature recorded during the photoperiod and during the night.

### Gases (including water vapour)

#### Water vapour (H_2_O)

*Description* Water vapour is the gaseous phase of water and can be produced from evaporation or boiling water, or from the sublimation of ice. Water vapour will condense onto another surface only when that surface is cooler than the dewpoint temperature, or when the water vapour equilibrium in the air has been exceeded. When water vapour condenses onto a surface, a net warming occurs on that surface. When water vapour evaporates from a surface, a net cooling occurs on that surface and/or the surrounding air. Condensation of water vapour on plant surfaces should be minimised as not to increase disease and pest issues. In plants, water loss from the stomata by evaporation and diffusion is termed transpiration. Excessive transpiration should be avoided as it can cause wilting.

*Measurements* Water vapour concentrations are typically measured in the air in/around the plant canopy. The amount of water vapour present in the greenhouse air can be expressed in different ways: while RH is a function of temperature, and is therefore a less desirable parameter to report, it is the parameter most often used to express the moisture content of greenhouse air. Many crops perform well when the RH ranges between 50 and 80%. Optimum vapor pressure deficit (VPD) values for most plant species range between 0.8 and 1.0 kPa. Small VPD values indicate humid conditions, making plant transpiration more difficult. Large VPD values indicate dry conditions, making plants susceptible to wilting. While many researchers report the relative humidity observed and/or maintained during their greenhouse experiments, it is more appropriate to use VPD because of its independence of temperature and because it is the driving force for transpiration.

#### Carbon dioxide (CO_2_)

*Description* CO_2_ is a trace gas comprising approximately 0.040% (by volume) of the Earth’s atmosphere. CO_2_ is toxic to humans at higher concentrations: 1% may cause drowsiness, above 5% is dangerous. During photosynthesis, plants, algae, and cyanobacteria absorb carbon dioxide, sunlight, and water to produce carbohydrates and oxygen as a waste product. During (dark) respiration, plants reverse this process and absorb oxygen and emit carbon dioxide. In greenhouses, the CO_2_ concentration can become the limiting factor for photosynthesis under high light conditions, necessitating adequate ventilation or active enrichment.

*Measurements* Carbon dioxide concentrations are typically measured in the air in or around the plant canopy. When detailed information about photosynthesis and/or respiration is needed, continuous measurements are necessary. Because accurate CO_2_ measurements require expensive equipment, a single sensor can be supplied with air samples from different locations and/or different greenhouse sections. These air samples can be supplied sequentially to the sensor with the help of a gas handling unit (sequencer). Readings can be stored using data acquisition systems (dataloggers). Sensor calibration can be accomplished using carefully prepared calibration gases. It is recommended that calibration is performed at no less than two concentrations: one at the low end and one at the high end of the measurement range. Depending on sensor type, temperature fluctuations (e.g. due to fluctuating exposure to solar radiation) can have an effect on measurements. In that case, sensors should be shielded from such fluctuations.

#### Oxygen (O_2_)

*Description* In plant nutrient solutions, the dissolved oxygen concentration can be an important indicator of the ability of the roots to respire. Similarly, in rooting media, the porosity (volumetric pore space fraction) is used as an indicator of the availability of O_2_ in the root zone.

*Measurements* Oxygen concentrations of the greenhouse air are not often of concern and are therefore almost never measured. When plants are grown in rooting media, the availability of oxygen to the roots is often assessed by determining the volumetric pore space fraction of the media. In nutrient solutions, the available oxygen to the roots is assessed by measuring the dissolved oxygen concentration. The dissolved oxygen concentration measurement is dependent on the nutrient solution temperature: the warmer the solution, the less oxygen it can contain. Thus, when the nutrient solution temperature is not controlled, frequent measurements are needed. A dissolved oxygen concentration in equilibrium with the oxygen concentration in the air above the solution is called the saturation concentration.

#### Effect of gaseous pollutants on plant growth and development

A variety of gaseous pollutants, some in very small quantities, have been reported to cause negative effects on plant growth and development, particularly in controlled environments when ventilation rates are limited. Many plastics, paints, sealants, glues, etc. release chemical compounds over time that can result in cumulative effects. Chemicals used in and around greenhouses can also release harmful compounds. High temperatures resulting from solar radiation or from inefficiencies in energy conversion (e.g. in electrical components) can increase the release of gaseous pollutants (e.g. NO_x_, SO_x_). Some plant species are highly sensitive to ethylene gas, a known plant hormone that is released, for example, from improperly operating combustion heating systems. People exhaling CO_2_ can significantly increase their surrounding CO_2_ concentration, particularly in small spaces. The most effective strategy to reduce the effect of gaseous pollutants is to minimise the use of materials that release (volatile) chemicals and to maintain adequate ventilation rates. Accurate measurements of (small) quantities of gaseous pollutants can require expensive equipment (e.g. gas chromatograph).

### Liquid water

#### Specifications

Water content of the rooting medium can be expressed in terms of volume, mass, filled pore space, matric (matrix) potential or water potential. The transport and retention of water in the growing media or soil is important to quantify.

The composition of irrigation water, including pH, alkalinity, electrical conductivity, total dissolved solids or salinity can have a profound effect on plant growth. The source of water can affect the composition/content of the water and consequently plant growth. In addition, water temperature can have an effect on plant growth as well as on the dissolved gases in the water, e.g. the dissolved oxygen content.

#### Measurements

In soilless culture systems, e.g. hydroponics, measuring properties of water is typically straight forward. In soil-based culture systems, the so-called flow-through method can be used to evaluate the characteristics of the aqueous fraction of the root environment. In each case, the assumption is made that the water characteristics are uniform throughout the system. This assumption may or may not be reasonable.

Electrical conductivity (S m^−1^) and pH should be recorded as well as the source of water, e.g. ground water, river/stream water, treated water (e.g. potable water from a local water company), deionised/distilled water, rainwater collected from the roof, including whether it includes recycled water from the greenhouse (e.g. condensate).

When soil water content or soil water potential is controlled, details about the control interval and the accuracy [proportion (%) of time the control parameter stayed within the control interval] of the control should be reported.

Irrigation water temperature should be recorded (especially when it varies during the day). The frequency of measurement should reflect the likelihood of variation. Delivery rate of irrigation water per square meter/pot, frequency (timing) and watering strategy should be reported. The fate of irrigation water should be reported, i.e. whether excess is allowed to run off or it is recirculated.

#### Effect on plant growth

Water is the largest component of plants, typically composing 70–95% of fresh plant mass and providing shape and rigidity by positive turgor pressure. Turgor pressure also drives cell elongation. Water is the transport medium for moving nutrients from the soil into the plant. Evaporation of water from the stomata provides cooling and prevents overheating of the leaves. The amount of water available can affect flower initiation, vegetative growth and fruit/leaf size.

#### Effect on plant development

A decrease in plant water content of 10% can greatly affect plant growth. Lack of water can reduce plant rigidity, reduce leaf expansion and result in irreversible damage to plant tissue.

### Nutrients

#### Description

Plants require inorganic nutrients (fertilisers) for proper growth and development [18, 19]. The typical plant contents, as proportion (%) of dry mass, for C, H, and O are approximately 41, 6, and 42%, respectively. All other essential nutrients (the remaining 11%) are typically provided to the rooting medium so they can be taken up by the roots. These essential nutrients are divided into two groups, the macro (or major) and the micro (or minor) nutrients based on the quantities needed by the plant. The macro nutrients include N, P, K, Ca, Mg, and S. The micro nutrients include Fe, Mn, Zn, Cu, B, Mo, Cl, Co, and Ni, and some of these are required only in extremely small amounts. When nutrients are provided (dosed) as part of the irrigation system, they need to be dissolved in water as ions (with a few exceptions, e.g. urea and boric acid). When nutrients are provided in solid form (e.g. as a slow release fertilizer incorporated into or placed on top of the rooting media), they need to be dissolved before they can be taken up by the roots. In some cases, foliar sprays are used to supply nutrients.

For liquid nutrient applications, in order to provide a mixture of nutrients with the correct concentrations, chemical compounds with known compositions can be dissolved in water. These compounds are commercially available in specific formulations and often include a complete breakdown of the nutrient composition. Chemicals can be mixed and dissolved into a concentrated stock solution, but some mixtures can result in precipitation (making those nutrients unavailable to the plants). This can be avoided by using two stock tanks each containing a different mixture of compounds that does not form a precipitate.

The pH of the aqueous fraction of the root environment has an effect on the availability of nutrients for uptake by the plant. Therefore, the pH is typically monitored regularly and controlled to within a relatively narrow range (e.g. ±0.2).

#### Measurements

Composition, dosing, and frequency of nutrient application should be recorded at the beginning of an experiment and at any time when rates are adjusted. Recommended measurements of the aqueous fraction of the root environment are described previously in the section entitled ‘Water’. When pertinent to the research, results from nutrient analyses should be reported, as well as the methods used to analyse the samples. Results from plant tissue analysis can be used to verify/adjust nutrient applications.

#### Effect on plant growth and development

When plants are not provided with the appropriate levels of nutrients, specific deficiency or toxicity symptoms may appear [19]. These symptoms are plant specific and often correlated with the level of deficiency or toxicity. These symptoms include, but are not limited to, discoloration, spotting, dead tissue, abnormal development, and stunted growth. The roles of various nutrients in plant growth and development have been studied extensively, but are not yet always fully understood.

### Greenhouse structure, growing system and environmental control system/strategy

#### Radiation

*Structure* The greenhouse structure and its orientation (typically expressed as the orientation of the ridge relative to due North) have a significant effect on the transmission of solar radiation [15, 20]. In particular, they determine the size, shape and movement of shadows across the growing area. Of the structural elements, the glazing material will have the most significant effect on the radiation flux and spectral distribution reaching the plant canopy. Therefore, greenhouse orientation and structural features, including the type of glazing material and whether a shading compound was added should be reported. Note that dust or dirt accumulation on the glazing can reduce light transmission, but this issue can be minimized by periodic cleaning. Since physical properties of some cladding materials can change over time, it is recommended to report on the age of the material and its general status. Where long-term data exist, it may be useful to report the average transmission of radiation as a proportion (%) of outside radiation.

*Growing system* The spatial uniformity of radiation emanating from supplementary lighting systems is less than that of solar radiation. Researchers need to quantify and report this uniformity so that its effect on plant growth and development can be assessed [15, 16]. The design and management of the growing system can also have an effect on the radiation environment: the design and location of the growing surface (e.g. benches, floor, and their surface characteristics), the presence of overhead equipment and/or plant material, and the number of re-spacings or relocations during the experiment.

*Environmental control system* The operation of a supplementary lighting and/or shading system will have a significant effect on the radiation environment. Therefore, operational procedures and control strategies should be reported. Design specifications, but preferably measured quantities (e.g. radiation flux, proportion of shading) should also be reported.

#### Temperature

*Structure* The greenhouse structure, in particular the type of cladding material (glass, rigid plastic or film), will have a significant effect on its heat transfer [5, 20]. Therefore, the type of greenhouse cladding material should be reported. Since the physical properties of some cladding materials change over time, it is recommended to report on the age of the material and its general status.

*Heating, ventilation, and cooling systems* Depending on the type of research and the usefulness of the data for other fields of study, it can be helpful to report on the type of heating system, its maximum capacity, and the control strategy implemented. In addition, information about additional measures implemented to improve energy distribution, such as perforated poly-tubes or horizontal airflow fans, should be reported. Typical greenhouse heating options include forced air (e.g. unit heaters, air furnaces), hot water (e.g. radiant floor and/or perimeter and overhead radiant pipe) and infrared systems. Typical ventilation options include natural (using sidewall and/or roof openings) and mechanical (using electrical fans and sidewall openings) systems. Typical (evaporative) cooling systems include pad-and-fan and high-pressure fog systems.

*Growing system* In order to provide a uniform growing environment for plants, it is important to assess the temperature distribution and variability. The number and capacity (at a specific static pressure) of all fans should be reported. The direction of airflow should also be reported in order to assess whether temperature gradients may be present. Despite increased airflow to improve the uniformity of temperature distribution throughout the greenhouse, the cropping system (particularly tall crops) can have a significant effect. A dense canopy can significantly reduce air movement and vigorous crop transpiration can (locally) reduce air temperature (converting sensible heat to latent heat). These factors should be taken into account and discussed when measuring and reporting air temperatures in greenhouse environments.

**Fig. 4:**
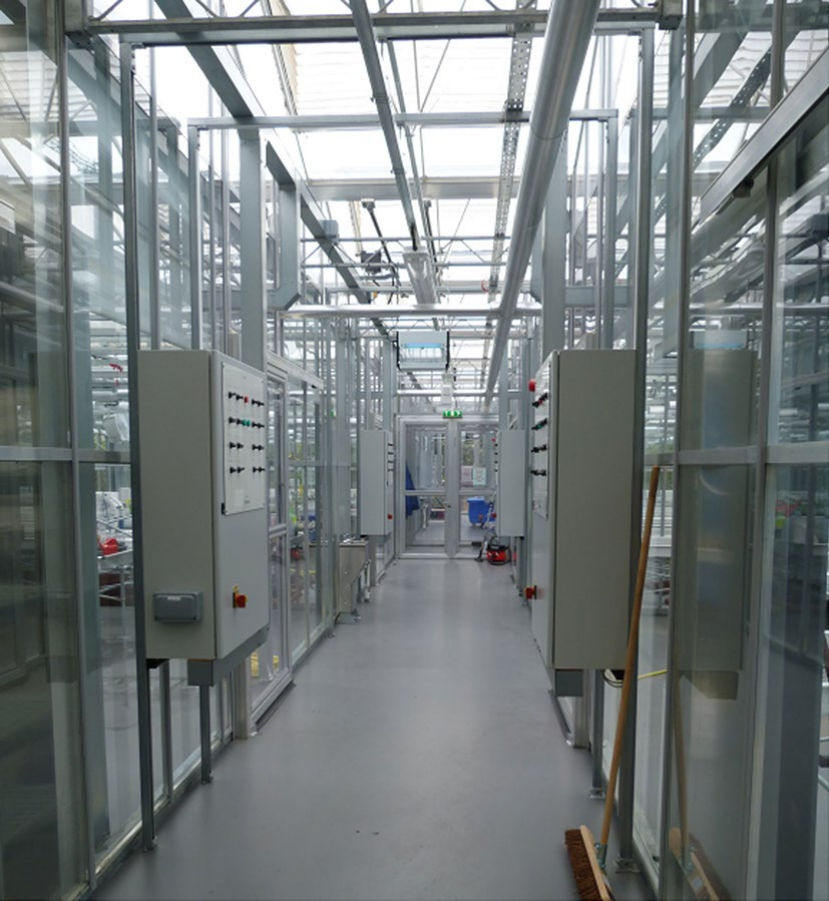
Greenhouse environmental control systems are diverse and frequently control algorithms are specific to the particular size, structure, orientation and location of the facility. Pertinent information on the system should be reported.

*Environmental control system* In order to save energy and reduce the plant respiration rate, many greenhouse operations implement different temperature set points during the day (photoperiod) and night [15, 16]. These temperature differences can also be used to control specific growth stages, e.g. stem elongation. Specific temperature regimes can also be used to vernalise some plant species (resulting in induction of germination or flowering). Different control strategies can be implemented to maintain greenhouse temperatures. Each control system and strategy uses a different method (control algorithm) to reach the desired outcome, resulting in different spatial and temporal temperature distributions. Some control strategies deliberately allow the temperature to fluctuate depending on outside conditions provided some daily average temperature can be achieved. Because of the effect of the various control systems and strategies, pertinent information about the systems and the control algorithms should be reported. In all cases, it is essential to report measured values and not set points (Fig. 4).

#### Gases (including water vapour)

*Structure* The tightness (leakiness) of the greenhouse structure will have an effect on the gas and water vapour concentrations inside. Outside wind conditions combined with a leaky greenhouse structure can have additional effect on gas and water vapour concentrations. The leakiness of a greenhouse structure can be evaluated through visual inspection for leaks or cracks, techniques to reveal airflow, or the concentration changes of deliberately released tracer gases. Cold glazing surfaces can result in condensation (and therefore, dehumidification), but the resulting water droplets can damage young plants when falling down instead of running into a collection system.

*Growing system* The growing system (e.g. bench designs that do or do not allow for vertical air movement) and the volume and density of the plant canopy can have a significant effect on the distribution and uniformity of the concentration of gases throughout the greenhouse. Air distribution systems (e.g. horizontal airflow fans or poly-tube distribution systems) can help distribute the air and thus improve uniformity. However, the uniformity of gas and water vapour distribution should be assessed spatially and temporally to ensure that the values reported were representative of the conditions experienced by all the plants during the experiments.

*Enrichment/removal systems*—*water vapour* Water vapour can be added or removed from greenhouse environments using humidifiers or dehumidifiers, respectively. Humidifiers produce water droplets of various sizes depending on their design and operation. Ideally, droplet size is minimised so as to speed up evaporation without wetting plant tissues. Air circulation is often used to enhance the distribution of the humidified air. Note that evaporative cooling, using either a pad-and-fan system or a high-pressure fog system, humidifies as well as cools the greenhouse air. Dehumidification using a refrigeration cycle to remove water vapour is an energy intensive and thus expensive approach. Instead, greenhouses can be dehumidified using the heat-and-vent strategy: heated air can hold more moisture that is then removed by ventilation. While energy is required for the heat-and-vent strategy, the benefits can outweigh the costs.

*Enrichment/removal systems*—*carbon dioxide* Carbon dioxide enrichment can be accomplished by distributing (pure) CO_2_ gas into the growing area. CO_2_ gas can be derived from different sources: from vapourised liquid CO_2_ stored in a refrigerated and pressurised container near the greenhouse site, or from burning natural gas and releasing the (scrubbed) exhaust gases inside the greenhouse. Uniform distribution is often accomplished by distributing the gas through small inflatable polyethylene tubes that have small openings along the length of the tubes. Typical target enrichment concentrations are 2–4 times the normal ambient concentration (approximately 750–1,500 µmol mol^−1^). Often, CO_2_ enrichment is suspended when the growing space needs to be ventilated to maintain the temperature set point. However, enrichment up to a certain minimum ventilation rate may still be beneficial and result in increased plant growth.

*Enrichment/removal systems*—*oxygen* Increasing the dissolved oxygen concentration of a nutrient solution can be accomplished by bubbling air or pure oxygen gas through the solution (e.g. using air stones). When using air, the maximum dissolved oxygen concentration attainable is by definition the saturation oxygen concentration (temperature dependent). When using pure oxygen gas, the maximum oxygen concentration attainable is approximately five times the saturation concentration (because air contains approximately 21% oxygen). Whether plants benefit from a super-saturated dissolved oxygen concentration in the nutrient solution has not been demonstrated, but using (pressurised) pure oxygen typically does not require additional energy to move the gas. When using air, compressors are often used and in that case care should be taken to ensure that the gas is not contaminated with lubrication particles that were inadvertently released from the mechanical components of the compressor.

#### Liquid water

*Growing system* When watering multiple pots or large areas, the expected or measured variance in water application should be reported as should whether watering is to excess or until soils or pots are at field capacity or even left standing in water. An indication of expected uniformity or reasons that good uniformity is to be expected should be provided. The choice of watering system can affect plant growth and development, especially because some systems can increase the risk of disease as a result of wet foliage (e.g. overhead spray systems).

*Environmental control system* Details about the control system if operated by e.g. water/rain sensor, leaf wetness sensor, tensiometer, soil conductivity sensor, lysimeter, or pot mass should be reported. The irrigation strategy should be reported, i.e. whether it is determined by a program based on sensor input, it is timed, it is dependent on light integral, or its timing and frequency are manually adjusted.

*Irrigation systems* Type of irrigation system, frequency and timing (length and time of day) of irrigation should be reported. Type and details may include:

- Manual (by lance/wand or hose): to surface, into saucer, watering when saucer dry, when soil surface dry, watering to run off.
- Drip system: Type and manufacturer of emitter, flow rate and operating pressure, number per pot, pressure compensated.
- Underground irrigation/seep hose: Type and manufacturer, depth, layout, delivery rate per meter.
- Overhead spray: Type and manufacturer, nozzle spray pattern, delivery rate.
- Flood bench (tide): Manufacturer (if applicable), periodicity of flooding, dwell time (period of flooding and to what depth).
- Wet bench: type of wet surface (sand type and porosity), capillary matting (type and water holding capacity) how water level maintained, details of any covering to reduce evaporation slope.
- Hydroponic or nutrient film technique (NFT) system: Details of the system should include growing media, flow rates, slope, oxygenation when relevant.
- Root misting systems: Details of system, volumes of water, size of root enclosure, droplet size used, whether misting is intermittent and, if so, its frequency.
- Weighing lysimeter: Details of system, plants watered to mass, either automatically or manually to maintain soil water potential.

Details of the irrigation system should include estimated delivery of water per application per pot or square metre, as appropriate, per day. In hydroponic systems water flow volumes over plant roots may be reported. Volumes or proportion (%) of the amount of recirculated water should be included.

#### Nutrition

*Growing system* Delivery method and the location of the release of nutrients in the root environment can have an effect on nutrient distribution and root growth. For example, top-down (e.g. overhead irrigation) or bottom-up (e.g. ebb and flood irrigation) will affect the movement of water and nutrients through the growing media and can result in zones with higher or lower nutrient concentrations. The type of growing media used can affect the availability of nutrients for uptake by the plants (e.g. certain nutrients will bind to clay particles, making them less available to plants). Hydroponic growing systems are typically designed to eliminate the possibility that the availability of water and/or nutrients becomes rate limiting. When growing in pots, researchers should be mindful that size, shape, material and even surface colour can influence root zone conditions and this should be considered when measuring and reporting plant responses.

*Environmental control system* Details about the nutrient control system (if used) should be reported. When data are available, the nutrient uptake rates should be reported. The temperature of the root zone will have an effect on the uptake rate of nutrients by roots, and is therefore more carefully controlled for some crop species. In some cases, the root zone temperature is actively controlled.

*Distribution systems* Liquid nutrient applications can be incorporated into the irrigation system (in that case, these systems are sometimes called fertigation systems). In most cases, small volumes of concentrated nutrient solutions are injected into the irrigation stream at a rate that ensures the proper dosing of the nutrients. Combinations of nutrients or individual nutrients can be injected this way, depending on the design and complexity of the nutrient delivery system. The injection can be passive (e.g. using a venturi system), or active (e.g. using an injection pump). Proper mixing is needed to ensure uniform distribution of the nutrients throughout the growing system. Adequate supply of nutrients throughout the rooting environment is critical since the uptake of nutrients by the roots will result in a gradient that could result in depleted regions.

## How to report experimental conditions

Here is an example of a report suitable for publication:

“The experiment was conducted in a research greenhouse section (located at 40°N, 90°W at 180 m elevation above sea level) with a floor area of 20 by 20 m and a bench area of 240 m^2^ elevated 0.75 m above the floor. The ridge of the greenhouse section was oriented North–South. The section was bounded to the East and West by identical, but differently controlled greenhouse sections and to the North by a central corridor. The eave (gutter) height was 2.5 m and the ridge extended 7.2 m above the floor. The greenhouse was mechanically ventilated with intake fans located in the South-facing side wall and capable of providing a maximum air flow rate of 0.06 m^3^ s^−1^ m^−2^ of floor area. Except for an entry door, the entire North-facing side wall was outfitted with 18.9 m^2^ of louvered ventilation opening and covered with an evaporative cooling pad and an insect screen designed to exclude white flies The greenhouse section was clad with 4-mm thick tempered glass and outfitted with an automated shade curtain with a manufacturer’s specified reduction in light transmission of 50%. The greenhouse was equipped with a supplementary lighting system powered by high-pressure sodium lamps and capable of providing a PAR flux of 200 (SD ±20) µmol m^−2^ s^−1^ at a distance of 1.75 m below the bottom of the luminaires. During the light period, the supplementary lighting system was turned on when the sunlight radiation flux was below 300 µmol m^−2^ s^−1^ at bench level, and turned off when the PAR flux reached 750 µmol m^−2^ s^−1^. A 15-min control delay was used to prevent excessive cycling of the supplementary lighting system. The greenhouse section was heated by a hot-water distribution system consisting of overhead pipes, perimeter pipes and tubes embedded in the solid concrete floor. Air temperature at canopy height was maintained at 20/15 (SD ±1)°C Day/Night. A 16-h photoperiod was imposed and centred on solar noon by operating the supplementary lighting system when no sunlight was available. The vapour pressure deficit (VPD) in the greenhouse section was maintained by operating a stand-alone humidifier or by employing the heat-and-vent control strategy. The VPD was continuously maintained at 1.0 (SD ±0.1) kPa. In the control hierarchy, VPD control had priority over temperature control. All sensors and instruments were calibrated annually following manufacturers’ specifications. The greenhouse section was outfitted with two horizontal airflow fans that operated continuously to improve mixing of the greenhouse air.

Starting on March 1, a geranium (*Pelargonium* × *hortorum*, cv. Maverick Red) crop was grown in the greenhouse section. Two cuttings were placed in a peat-vermiculite soil-less mixture (1:1 sphagnum peat:vermiculite by volume, and amended with 0.5 kg of ground dolomitic limestone per m^3^ of mixture) contained in 0.75 L plastic pots. Each pot received a dose (at an equivalent rate of 3 kg m^−3^) of slow release fertiliser (10-10-10) and was irrigated with a drip system fed with deionised water. The initial pot density on the benches was 64 m^−2^ for the first 6 weeks, followed by a density of 36 m^−2^ for the remainder of the experiment. The experiment was terminated on May 15.

This reporting example describes only those parameters that the researcher deemed important for the particular experiment conducted. While the information reported may be sufficient for the intended audience (e.g. fellow researchers doing similar experiments), other researchers may find the report insufficient, in particular when they study environmental parameters not specifically discussed in the report. Therefore, researchers are strongly encouraged to consider carefully which parameters to measure and report on and the impact this decision may have on the usefulness of their work to other researchers including those not directly involved in their field of interest.

## Endnotes

^a^The information presented was compiled by the International Committee on Controlled Environment Guidelines (ICCEG). The ICCEG was sponsored by and this paper published for the UK Controlled Environment Users’ Group (CEUG; http://www.ceug.ac.uk/), the North American Committee on Controlled Environment Technology and Use (NCERA-101; http://www.controlledenvironments.org/) and the Australasian Controlled Environment Working Group (ACEWG).
